# As a prognostic biomarker of clear cell renal cell carcinoma RUFY4 predicts immunotherapy responsiveness in a PDL1-related manner

**DOI:** 10.1186/s12935-022-02480-7

**Published:** 2022-02-08

**Authors:** Daojia Miao, Jian Shi, Zhiyong Xiong, Wen Xiao, Xiangui Meng, Qingyang Lv, Kairu Xie, Hongmei Yang, Xiaoping Zhang

**Affiliations:** 1 Department of Urology, Union Hospital, Tongji Medical College, Huazhong University of Science and Technology, Wuhan, 430022 China; 2 Institute of Urology, Union Hospital, Tongji Medical College, Huazhong University of Science and Technology, Wuhan, 430022 China; 3 Department of Pathogenic Biology, School of Basic Medicine, Huazhong University of Science and Technology, Wuhan, 430030 China

**Keywords:** Clear cell renal cell carcinoma, Immune infiltration, Immunotherapy, *PD-1* blockade, Prognostic signatures

## Abstract

**Background:**

Clear cell renal cell carcinoma (ccRCC) is one of the most lethal malignancies in the urinary system and the existing immunotherapy has not achieved satisfactory outcomes. Therefore, this study aims at establishing a novel gene signature for immune infiltration and clinical outcome (overall survival and immunotherapy responsiveness) in ccRCC patients.

**Methods:**

Based on RNA sequencing data and clinical information in The Cancer Genome Atlas (TCGA) database, we calculated proportions of immune cells in 611 samples using an online tool CIBERSORTx. Multivariate survival analysis was conducted to determine crucial survival-associated immune cells and immune-infiltration-related genes (IIRGs). Next, the clinical specimens and common renal cancer cell lines were applied to confirm IIRGs expression at protein and RNA levels. Finally, functional enrichment analyses and siRNA technology targeted to *RUFY4* were implemented to verify its function of predicting immunotherapy response.

**Results:**

Follicular helper T cells (TFHs) and Regulatory T cells (Tregs) were highly infiltrated in the tumor microenvironment (TME) and their relative proportions were independent prognostic factors for patients. Among IIRGs of TFHs and TREGs, *RUFY4* was found to be highly activated in tumor microenvironment and its co-expression network was enriched in *PDL1/PD1* checkpoint pathway in cancer. Additionally, knockdown of *RUFY4* led to the decline of *PDL1* and proliferation ability in ccRCC cell lines.

**Conclusion:**

TFHs and Tregs were considered as prognostic biomarkers and *RUFY4* was an immunotherapeutic predictor of ccRCC patients in a *PDL1*-Related manner.

**Supplementary Information:**

The online version contains supplementary material available at 10.1186/s12935-022-02480-7.

## Background

Global statistics indicate that kidney cancer accounts for approximately 2.2% of all cancers, with 1.8 million new cases and 175,100 deaths recorded in 2018 [1]. Clear cell renal cell carcinoma (ccRCC) is the predominant subtype among all kidney cancer subtypes, representing over 70% of all RCC cases [2]. In the past decade, antiangiogenic drugs targeting vascular endothelial growth factor (*VEGF*), mechanistic target of rapamycin (mTOR) inhibitors, and immune checkpoint inhibitors (ICI) have rapidly expanded treatment options for metastatic ccRCC [3]. Though great improvements in managing ccRCC have been achieved via specific cellular and molecular targets, the prognosis of patients is still unsatisfactory.

The interaction between cancer and immune cells in the tumor microenvironment (TME) has been a new hallmark of cancer since 2011 [4]. As an indispensable part of the TME, immune infiltration cells have been shown to play crucial roles in the occurrence and development of tumors [5]. In previous studies, ccRCC has been considered as a highly immune-infiltrated tumor and infiltration of different immune cells was correlated with distinct clinicopathologic features [6, 7]. Studies by Ueda [8] and Mikami [9] have suggested that immune-infiltrated-related genes (IIRGs) are associated not only with the response to immunotherapy but also with the clinical outcome of ccRCC patients. However, their accuracy and specificity in predicting sensitiveness to immunotherapy and prognosis of ccRCC remain problematic. Therefore, to improve the survival time and life quality of ccRCC patients and provide worthy information to conduct precise individual treatment, novel immune predictors and prognostic indicators are urgently needed.

RUN and FYVE domain-containing proteins (RUFYs), consisting of an N-terminal RUN domain and a phosphatidylinositol 3-phosphate (PI3P)-interacting C-terminal FYVE domain, encompass four genes named *RUFY1-4* [10]. Briefly, *RUFY1-2* contain a RUN domain and a FYVE domain, separated by two coiled coil domain and both of them are identified as downstream effectors of Etk protein kinase [10]. *RUFY3,* the smallest of the RUFY proteins with only a RUN domain, is mostly expressed in neurons [11]*. RUFY4* is atypical among the RUFY family members, since it only holds one coiled coil domain [12]. Currently, RUFYs have taken center stage in the field of oncology [11]. *RUFY1* has been shown to interact with podocalyxin-like (*PODXL*) protein, an ion exchanger regulatory factor in the membrane protein complex associated with poor prognosis of different cancers [13, 14]. Shin [15] suggested *RUFY2* as one of the extremely frequently mutated genes in colorectal cancer. Besides, researches by Zheng [16] and Staubitz [17] showed that *RUFY2* participated in tumorigenesis in lung adenocarcinoma and papillary thyroid carcinoma. *RUFY3* is the final well-known protein in the RUFY family and its dysregulation is implicated in the growth, invasion, and metastasis of lung adenocarcinoma [18] and colorectal cancer [19]. Although *RUFY4* is reported to be the first molecule that affects autophagy and endosome dynamics in a subset of immune cells and that *RUFY4* is an important factor that defines the nature of the response of cells to the direct immune environment [12], there is no strong evidence that *RUFY4* is relevant to any type of cancer.

Based on The Cancer Genome Atlas (TCGA) database, the present study quantified the composition of immune cells in ccRCC and figured out two types of immune cell that possess prognostic ability of ccRCC. Furthermore, we identified *RUFY4*, as a novel IIRG, that could simultaneously predict patient prognosis and immunotherapy responsiveness in a *PDL1*-related manner. Our study also offered a novel method for researchers to creatively tap into immunotherapeutic cells and gene signatures for other cancers.

## Materials and methods

### Data source and cleaning

The ccRCC RNA-Seq data were obtained from TCGA database via the Data transfer tool. Samples information and clinical data which consist of gender, age, clinical TNM stage, histopathological grade, survival time, etc. were directly collected from the website. Then, two gene expression matrices of COUNT and FPKM (Fragments Per Kilobase of exon model per Million mapped)) and one clinical information table were constructed through R software (version: 64 4.0.3). Meanwhile, Yusenko renal datasets from the Oncomine (https://www.oncomine.org/) database and GSE126864 from GEO (Gene Expression Omnibus) database were also analyzed in this study. The analytical procedures were shown in Additional file 1: Fig. S1.

### Establishment of immune infiltration landscape

CIBERSORTx is an online tool to accurately infer cell type abundance from RNA profiles of intact tissues [20]. This study conducted CIBERSORTx analysis online (https://cibersortx.stanford.edu/) according to the following parameters:

*sigmatrix: LM22.update-gene-symbols.txt, perm: 500, verbose: TRUE, rmbatchBmode: TRUE* *,* *QN* *:* *FALSE*

From calculation results of CIBERSORTx, 609 samples were selected with p ≤ 0.05 and each sample was equipped with the relative proportion matrix of 22 immune cell types. Then R packages “pheatmap”, “barplot” and “vioplot” were installed in R software to establish the immune infiltration landscape.

### Identification of survival-associated immune cells

On basis of the proportions of 22 immune cells, Kaplan–Meier (K-M) analysis of overall survival rate was performed. Among K-M survival analysis, the cut-off was set as the upper and lower quartiles and statistically significant means that p-values were less than 0.05. Univariate survival analysis was used to verify the prognostic predictive effect of known factors, such as age, gender, T stage, M stage, etc. Additionally, this study created a multivariate model which was adjusted for those factors whose hazard ratio (HR) > 1. By setting the cut-off as the median of relative proportion, 537 samples were distributed into high and low groups. Then the proportion of each immune cell was added as a new binary variable for survival analysis.

### Identification of key genes related to immune infiltration

Among genes that are highly expressed in tumors, this study searched for genes that are closely related to the high degree of immune-infiltration of immune cells. Also, the same method was used to identify the low expressed key genes. R package “VennDiagram [21]” was installed on R software to visualize these results. The receiver operating characteristic (ROC) curve of each key gene was used to assess the capability of distinguishing from patients for healthy individuals according to areas under this curve (AUC).

### Construction of RUFY4 co-expression network

The cBioPortal offers researchers online assistance for studying multidimensional cancer genomics data [22]. To construct the co-expression network of *RUFY4*, genes with a Spearman correlation index > 0.55 were added to co-expression network.

### Functional enrichment analysis

This study carried out functional classification and annotation of immune-associated genes on the website DAVID [23] by the methods of GO and KEGG analysis. The cut-off of p-value was 0.05. R packages “ggplot2” were used to visualize the top 10 terms of GO analysis and KEGG pathway analysis. Then, Cytoscape [24] was also applied to construct the pathway cross-talk network via a “Cluego” plug-in. Gene set enrichment analysis (GSEA) was performed by Windows desktop program v4.1.0. The GSEA result of *RUFY4* was therefore conducted on all known genes ranked by enrichment scores from most positive and most negative. 1000 random sample permutations were carried out.

### Prediction of immunotherapy response

ImmuCellAI was used to predict the response of Immune checkpoint blockade (ICB) therapy with the ICB response prediction being checked based on gene expression matrix [25]. This study conducted ICB prediction analysis online (http://bioinfo.life.hust.edu.cn/ImmuCellAI#!/analysis) based on the compositions and proportions of immune cells of patients in TCGA.

### Cell culture and reagents

The human renal cell carcinoma cell line A498, 786O, CAKI, OSRC and control cell line HK2 were obtained from the American Type Culture Collection (ATCC, USA). Cells were cultured in with Dulbecco's modified eagle medium (DMEM, Gibco, USA) supplemented with 10 percent fetal bovine serum (FBS, Gibco, USA) and were cultured in the incubator at 37 °C, 5% carbon dioxide.

### RNA interference

Small interfering RNA for *RUFY4* was transfected in 786O and CAKI cell lines using Lipofectamine 6000 (Beyotime, China), respectively. The siRNA sequences for *RUFY4* (GenePharma, China) were followings:

siRNA#1:5′–3′ CAAGGUCACCAAAGACCUAAG

siRNA#2:5′–3′ GGAGAAUCCACAAGUGCAAAC

siRNA#3:5′–3′ GCAGAGGGUCAGAGAACAACA

Cell lysates and total RNA were collected 72 h after the transfection to verify knockdown efficiency by western blot and qPCR.

### Tissue samples

16 pairs of human ccRCC tissues and adjacent normal tissues were collected from Department of Urology, Union Hospital, Tongji Medical College (Wuhan, China) in 2020. This process had fully informed consent of the patients. And this study was approved by the Institutional Review Board of Huazhong University of Science and Technology. The license number of the ethical review for the study was S1892.

### RNA isolation and real-time PCR analysis

The Magzol reagent (Thermo, Massachusetts, USA) was used to extract total RNA of tissues and cell lysates. 500 ng of total RNA from tissue and cell were applied for reverse transcription. qPCR analysis was conducted (LightCycler 480II; Roche, Basel, Switzerland) with the Hieff^®^ qPCR SYBR Green Master Mix (11201ES03, Yeasen, China). Samples were normalized by Glyceraldehyde-3-Phosphate Dehydrogenase (*GAPDH*).

*GAPDH*: Forward 5′-CCAGAACATCATCCCTGCCT-3′
  Reverse 5′-CCTGCTTCACCACCTTCTTG-3′
*RUFY4*: Forward 5′-ACGCCAAGAAGACATCCTGG-3′
  Reverse 5′-CTCTGACCCTCTGCAACCAG-3′

### Western blotting assays

The protein of cells and tissues was extracted by radio-immunoprecipitation assay (RIPA) protein lysis buffer (Beyotime, China) with protease inhibitor cocktail (Beyotime, China) and Phenylmethanesulfonylfluoride (PMSF, Beyotime, China). 30 µg of protein was subjected to sodium dodecyl sulfate–polyacrylamide gel electrophoresis (SDS-PAGE) gel. The proteins were then separated by gel electrophoresis and transferred to polyvinylidene fluoride (PVDF, Roche, Basel, Switzerland) membranes. 5% nonfat dried skimmed milk was used to block the membranes for 1.5 h at room temperature. Then, the membranes were incubated overnight with primary antibodies.

*RUFY4*, LS‑C307570 LSBio, USA, dilution 1:2000

*PDL1*, A1645 ABclonal, China, dilution 1:1000

*β-actin*, AC026 ABclonal, China, dilution 1:100,000

Finally, the membranes were washed and incubated in blocking buffer with secondary antibodies (Anti-Rabbit AS014 ABclonal, China; Anti-Mouse AS003 ABclonal, China) for 2 h before detection.

### Cell viability assays

Each 96‐well plate was plated with 2000 cells. The proliferation rate of cells was detected using the cell counting kit (CCK-8, Yeasen, China). 110 μL CCK8 solution (10 μL CCK8:100 μL medium) were added to each well and the 96-well plate was incubated in dark for 1 h. Cell viability was assessed at 0, 24, 48, 72 and 96 h upon treatments by NanoDrop 2000 spectrophotometer (NanoDrop Technologies, USA) at 450 nm.

### Transwell assays

For migration and invasion assays, cells were cultured in serum-free medium for 24 h. Then, cells were plated in the top chamber of transwell chamber (REF3422, Corning, USA) and cells were allowed to invade through the Matrigel (Corning, USA, dilution 1:8) or not. With or without Matrigel were used for invasion and migration assay. After 24 h, cells invading the lower surface of the chamber membrane were fixed in 100% methanol. Then, cells were stained with 0.05% crystal violet and 10 fields were randomly photographed for counting. More details were described in previous study [26].

## Results

### A unique immune infiltration landscape was established for ccRCC

**Fig. 1:**
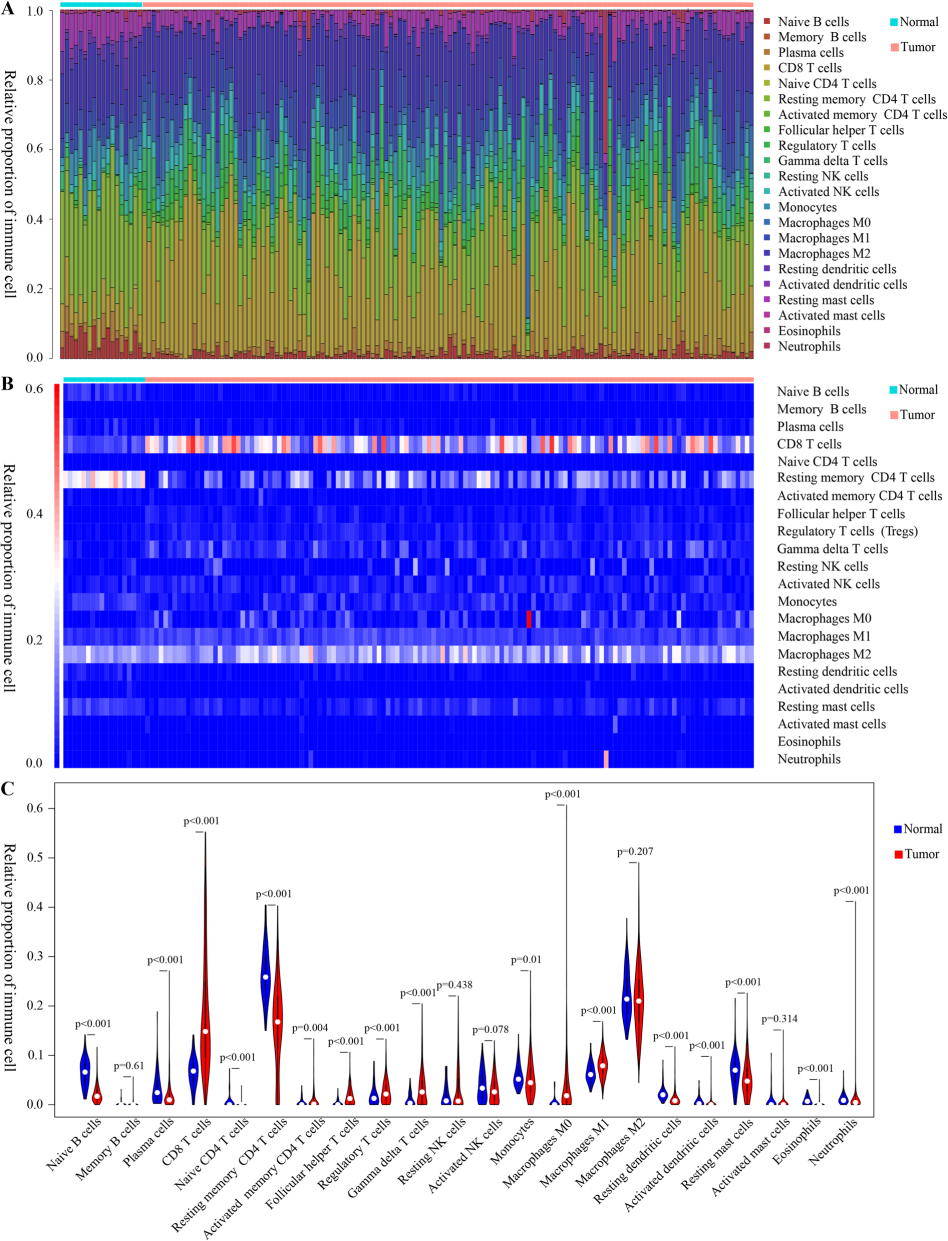
Establishment of immune-infiltration landscape. **A **The relative proportions of immune cells in the samples from TCGA. Each column represents a sample, and each column with a different color and height indicates the relative proportions of immune cells in this sample. **B **The heatmap of 22 immune cells in tumor and adjacent normal tissues. Each row represents one type of immune cell and the color of each small square represents the content of immune cells. **C **The difference of 22 immune cell relative proportions between the ccRCC and control groups in TCGA. The blue violins represent adjacent normal tissues while the red violins represent tumor tissues. Wilcoxon-test were used in statistical analysis. p valves were displayed in Figures

When the RNA-seq matrix derived from 611 ccRCC samples were screened by the CIBERSORTx algorithm, the differences of immune infiltration between ccRCC and adjacent normal tissues were comprehensively tracked out. The proportions of 22 immune cells in ccRCC and adjacent normal tissues were shown in Fig. 1A. The heatmap of the 22 immune cells was shown in Fig. 1B. To further examine the difference of immune infiltration cells, we generated a violin plot to quantitatively describe the differences based on tumor tissues (537 samples) and adjacent normal tissues (72samples). According to the violin plot (Fig. 1C), naïve B cells, plasma cells, CD8 T cells, naïve CD4 T cells, resting memory CD4 T cells, activated memory CD4 T cells, follicular helper T cells (TFHs), regulatory T cells (Tregs), gamma delta T cells, monocytes, M0 macrophages, M1 macrophages, resting dendritic cells, activated dendritic cells, resting mast cells, eosinophils and neutrophils presented different relative proportions and the differences were statistically significant. Immune cell types, such as CD8 T cells, were markedly highly infiltrated in tumor tissues. These results showed that the proportions of immune-infiltrated cells might help researchers to distinguish ccRCC from normal individuals.

### TFHs and Tregs were identified as survival-associated immune cell

**Fig. 2:**
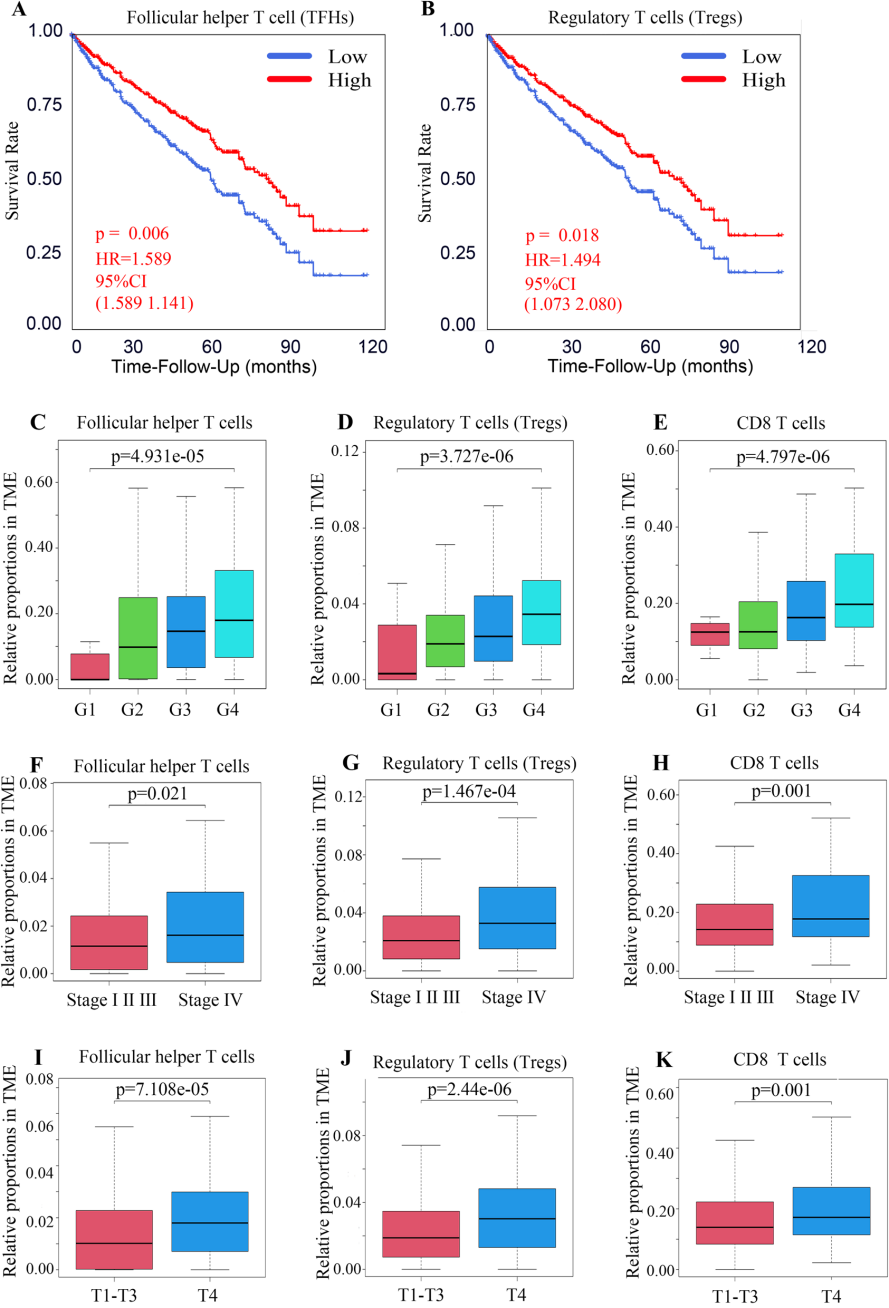
TFHs and Tregs were identified as survival-associated immune cell. **A**, **B **The multivariate survival analysis for the relative proportions of immune cells. The red line indicates a high proportion group of immune cells, and the blue line indicates a low proportion group of immune cells. HR means hazard ratio and CI means confidence interval. p-valve were on Figures. **C**–**K **The relationship between the relative proportions of immune cell and G grade, clinical stage and T stage. The ordinate represents the proportion of immune cells and the horizontal line inside the box represents the median value of immune cell proportions. Kruskal-test and Wilcoxon-test were used in statistical analysis and p-valves were on Figures

In the previous step, 17 immune cell types were obtained based on their differences in immune infiltration between tumor and adjacent normal tissues. Here, the correlation among the proportions of the 17 immune cell types and overall survival (OS) rates were analyzed via Kaplan–Meier curves. The results in Additional file 2: Fig. S2A–H showed that the proportions of 8 immune cells were related to the OS rates, including activated memory CD4 T cells, resting memory CD4 T cells, CD8 T cells, M0 macrophages, M1 macrophages, Tregs, TFHs, and neutrophils. Notably, TFHs and Tregs had the highest hazard ratio (HR) values ​​of 1.59 and 1.62, respectively, while resting memory CD4 T cells and neutrophils were negatively correlated with the OS rate. To further understand the clinical predictive value of survival-associated cells, the spearman correlation analysis was conducted among the relative proportion of immune cells and clinical attributes. As shown in Fig. 2C–K and Additional file 3: Fig. S3A–C, the proportions of TFHs, Tregs and CD8 T cells elevated with the increase of histopathological grade and TNM stage. The results of other immune cells were shown in Additional file 3: Fig. S3D–G. Next, multivariate survival analysis was conducted to assess the prognostic value of the 8 immune cells and a clinicopathologic parameter was chosen as OS rates for ccRCC patients (Table 1). Relative proportions of TFHs and Tregs were independent prognostic factors for OS, after adjustment for known risk factors such as age, clinical stage, T stage, M stage, and histopathological grade (Fig. 2A, B). Survival analyses indicated that a higher immune infiltration of TFHs or Tregs was associated with inferior OS. OS curves based on TFHs or Tregs proportions in ccRCC tumor tissues were distinctly disjoined. Therefore, TFHs and Tregs held much appeal for this study.

**Table 1:** Multivariate analyses of immune cell fraction and patient survival

Variable	HR	95% CI		p-valve
		Low	Up	
Age (years)				
≤ 60 (n = 267)				
> 60 (n = 270)	1.71	1.231	2.374	0.001
Gender				
Female (n = 186)				
Male (n = 351)				> 0.05
Clinical stage				
C1–C3 (n = 452)				
C4 (n = 82)	14.832	3.417	64.394	< 0.001
CX (n = 3)				
T stage				
T1 and T2 (n = 348)				
T3 and T4 (n = 189)	1.689	1.150	2.482	0.008
M stage				
M0(n = 447)	0.189	0.044	0.811	0.025
M1 (n = 80)				
MX (n = 10)				
G stage				
G1 and G2 (n = 249)				
G3 and G4 (n = 281)	1.797	1.212	2.665	0.004
GX (n = 7)				
Tregs				
Low (n = 268)				
High (n = 269)	1.494	1.073	2.080	0.018
TFHs				
Low (n = 268)				
High (n = 269)	1.589	1.141	2.212	0.006

Multivariate models were adjusted for T, M classification, age, and gender

Hazard ratio, estimated from Cox proportional hazard regression model

Confidence interval of the estimated HR

### IIRGs of Tregs and TFHs participated in several overlapped biological processes

**Fig. 3:**
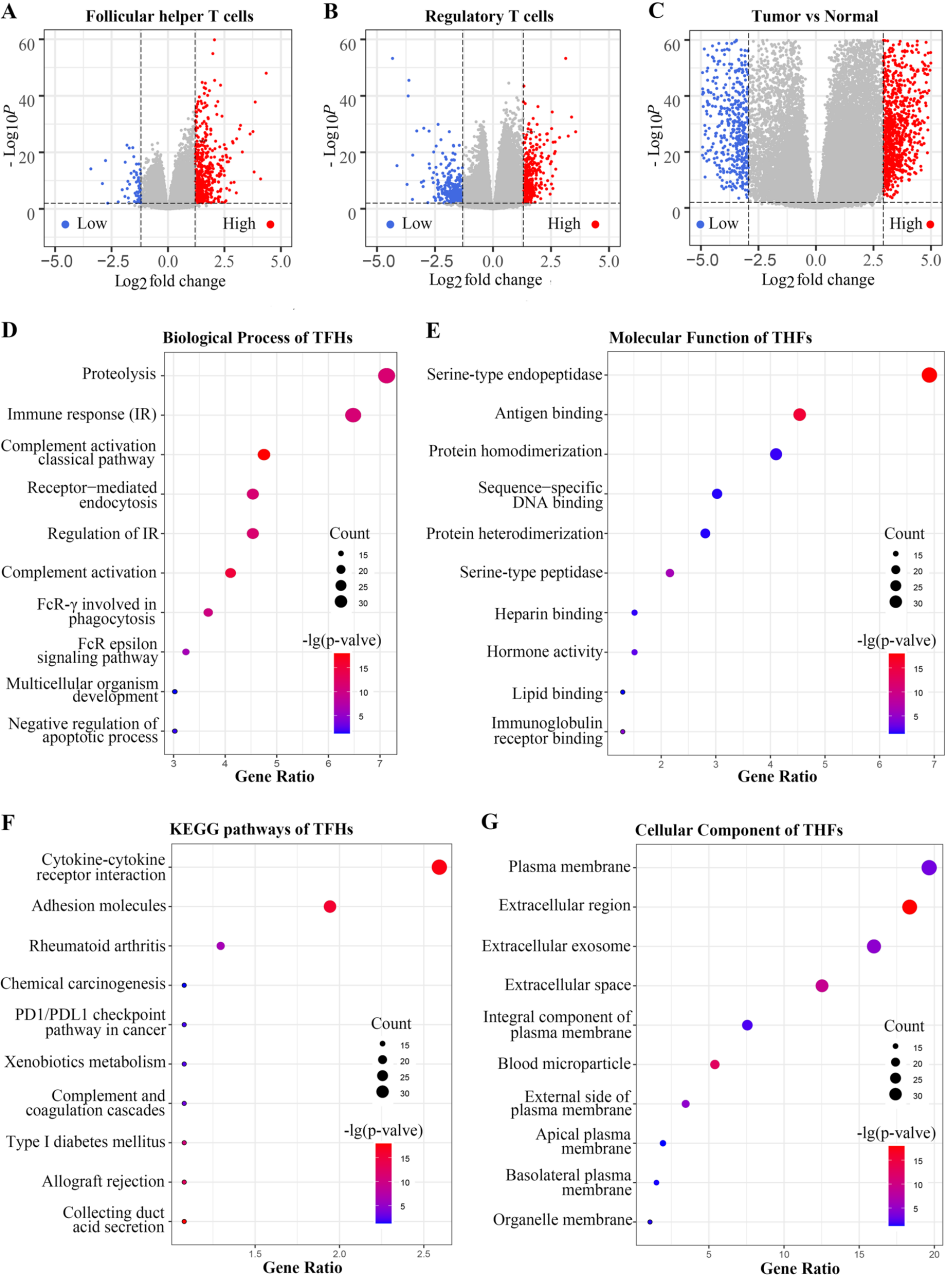
IIRGs of Tregs and TFHs participated in several overlapped biological processes in TME. **A **DEGs between high TFHs-infiltration group and low TFHs-infiltration group. **B **DEGs between high Tregs-infiltration group and low Tregs-infiltration group. **C **DEGs between ccRCC and adjacent normal tissues. Each red dot shows an upregulated gene and each blue dot shows a downregulated gene. DEGs means different expression genes. **D**–**G** the enrichment analysis results of IIRGs at biological processes, cellular components, molecular functions, and KEGG levels. The top 10 results of each term are shown, and the color indicates the statistical significance and the size indicates the number of genes enriched for each result. IIRGs is the abbreviation of immune-infiltration-related genes

This study obtained 680 genes that had significantly different expression levels between high and low proportions of TFHs in the TME, 620 of which presented much higher expressions (Fig. 3A). As for Tregs, 676 differentially expressed genes were identified, where 353 genes were related to higher immune infiltration (Fig. 3B). After uploading these genes to the DAVID website, the top 10 items of TFHs in Gene Ontology (GO) and Kyoto Encyclopedia of Genes and Genomes (KEGG) analyses were obtained (Fig. 3D–G). The biological process of the GO analysis demonstrated that TFHs might be involved in the cellular protein metabolic process and immune response (Fig. 3D). The cellular component showed that these genes were enriched in extracellular exosome and plasma membrane (Fig. 3G). The molecular function of these genes was gathered in serine-type endopeptidase activity, antigen binding and sequence-specific DNA binding (Fig. 3E). The KEGG analysis suggested that cytokine-cytokine receptor interaction was the dominant pathway and *PDL1*/*PD1* pathway was also enriched (Fig. 3F). As for Tregs, cellular protein metabolic process at the biological process level, extracellular exosome at the cellular component level and serine-type endopeptidase activity at molecular function level were the most crucial enrichment items (Additional file 4: Fig. S4A–D). KEGG pathway analysis results were largely similar to TFHs results. Collectively, these results showed that the two crucial immune-associated cells had a considerable overlapped function, implying that the two cells might participate in the same biological process and there might be cross-talks within the TME.

### Upregulated-RUFY4 was a prognostic biomarker of ccRCC

**Fig. 4:**
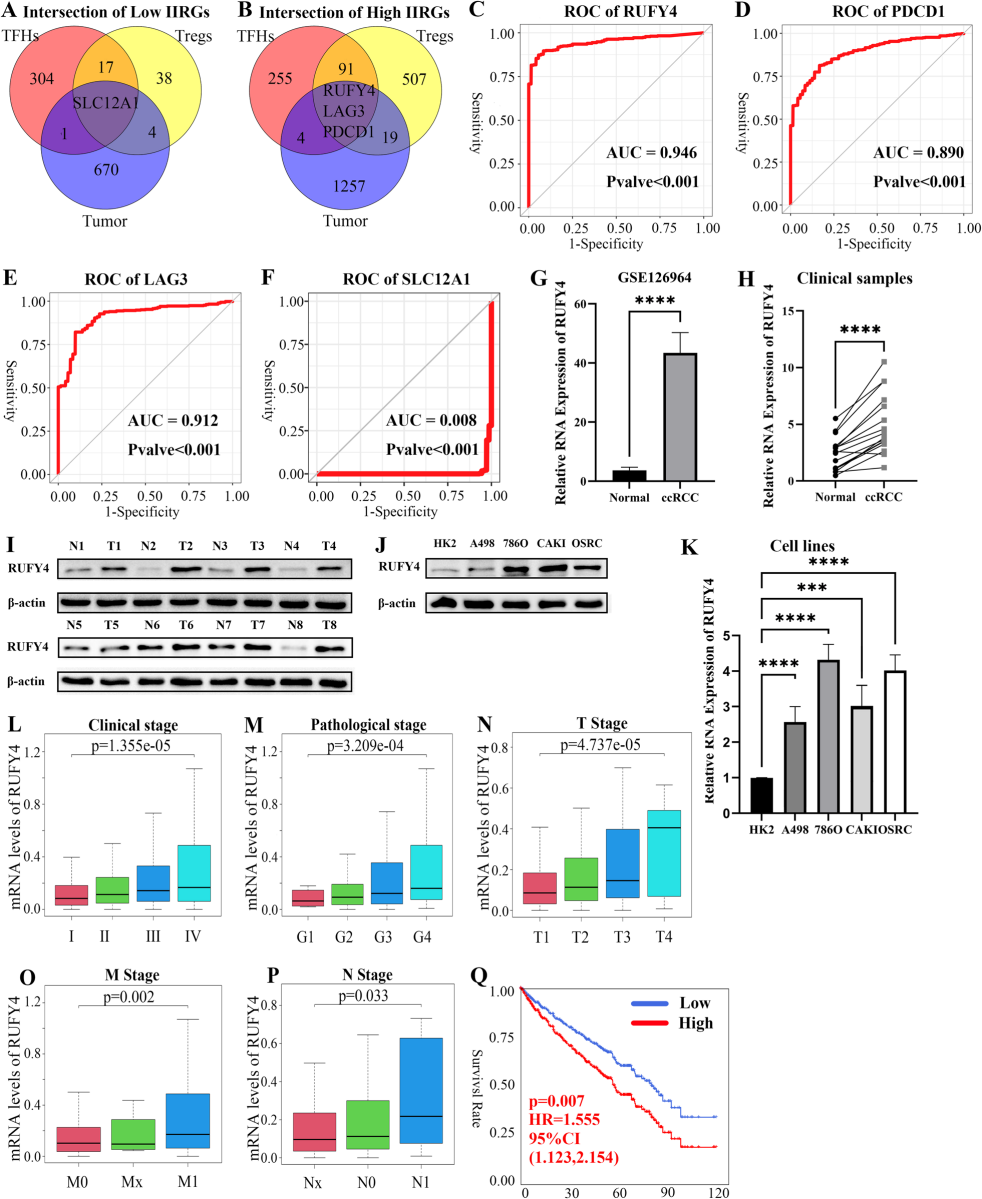
Upregulated-RUFY4 was a prognostic biomarker of ccRCC. **A **Using Venn plot to pick up genes that are low-expressed in tumors and related to low-density immune-infiltration. **B** Contrary to A, the figure shows genes that are highly expressed in tumors and related to high-density immune-infiltration. **C**–**F** Receiver operating characteristic (ROC) curve of *RUFY4*, *PDCD1*, *LAG3*, and SCL12A1. ROC curves indicate the capabilities of picking up ccRCC patients from the public. AUC means the area under a curve. p-valves were on Figures.** G **The mRNA levels of *RUFY4* in 55 ccRCC tissues and 11 paired adjacent normal tissues in ccRCC based on data from the GEO database. t-testp < 0.0001. **H **The mRNA levels of *RUFY4* in 16 pairs of ccRCC tissues and adjacent normal tissues. t-test, ****p < 0.0001. **I** The protein levels of *RUFY4* in 8 ccRCC tissues and 8 paired tissues in ccRCC. **J** The protein levels of *RUFY4* in HK2 and RCC cell lines. **K **The mRNA levels of *RUFY4* in HK2 and RCC cell lines. t-test, ****p < 0.0001, ***p < 0.)01. **L**–**P **The relationship between the expression level of* RUFY4* and clinical stage, G grade, T stage, M stage, and N stage. Kruskal-test and Wilcoxon-test were used in statistical analysis. p-valves were on Figures. **Q **The multivariate survival analysis for the expression level of *RUFY4*. The red line indicates a high expressing group of *RUFY4*, and the blue line indicates a low expressing group. HR means hazard ratio and CI means confidence interval. p-valves were on Figures

Three gene sets, including genes related to low immune infiltration of Tregs, low immune infiltration of TFHs, and genes low-expressed in tumor tissues (shown in the previous Fig. 3C), were submitted to the “VennDiagram” Package on R software. As shown in Fig. 4A, *SLC12A1* was closely related to the low degree of immune infiltration of the two crucial immune cells and it was silenced in tumor tissues. Remarkably, after narrowing the search scope to genes that were highly expressed in tumor tissues, *LAG3*, *PDCD1*, and *RUFY4* were found to be associated with the high degree of immune infiltration of Tregs and TFHs. From Fig. 4B, it could be observed that 94 genes were not only associated with the increased immune infiltration of Tregs but also with the immune infiltration of TFHs. The ROC results of the aforementioned 4 key genes, including one downregulated gene *SLC12A1* and 3 upregulated genes *LAG3*, *PDCD1* and *RUFY4*, are presented in Fig. 4C–F. *RUFY4* had a relatively higher AUC (area under the curve = 0.946) than the other key genes (0.890, 0.912, 0.008), suggesting that it had a more accurate diagnostic value for ccRCC. Though *LAG3* also had a significant AUC, it had little prognosis prediction valve (Additional file 5: Fig. S5A). Therefore, *RUFY4* was considered as the key gene in the TME related to the high immune infiltration of TFHs and Tregs (Table 2). Besides, *RUFY4* had an activated expression in tumor tissues, hence could accurately and sensitively distinguish between normal and ccRCC patients. To verify these results, the high throughput sequencing data in GSE126964 were downloaded from GEO database. From Fig. 4G, it could be demonstrated that *RUFY4* was also upregulated in ccRCC. The same results were obtained from the collected clinical samples (Fig. 4H). Correspondingly, changes of the protein level were consistent with the RNA level of *RUFY4* (Fig. 4I). Additionally, common RCC cell lines, such as 786O [27] and CAKI [27], presented higher protein and RNA levels than HK2 that is an immortalized proximal tubule epithelial cell line from normal adult human kidney (Fig. 4J–K). Moreover, the immunohistochemical results from the Human Protein Atlas [28] (HPA is a resource for many areas of biomedical research, including protein science and biomarker discovery) also showed that *RUFY4* was highly expressed in renal cancer (Additional file 5: Fig. S5B). Next, this study determined the correlation between *RUFY4* and ccRCC clinical attributes. *RUFY4* expression levels were consistently correlated with ccRCC clinical stage (Fig. 4L), histopathological grade (Fig. 4M), T stage (Fig. 4N), M stage (Fig. 4O), N stage (Fig. 4P). Survival analysis revealed that the increased expression of *RUFY4* was associated with inferior OS (Fig. 4Q). In summary, these results confirmed that *RUFY4* was a new prognostic biomarker of ccRCC.

**Table 2:** Spearman corelation analyses of immune cell fraction and RUFY4&LAG3

	RUFY4		LAG3	
Immune cells	Spearman r	p-value	Spearman r	p-value
Naïve B cells	− 0.505	****	− 0.596	****
Memory B cells	0.024	ns	0.003	ns
Plasma cells	− 0.071	ns	− 0.027	ns
CD8 T cells	0.528	****	0.821	****
Naïve CD4 T cells	− 0.212	****	− 0.207	****
Resting memory CD4 T cells	− 0.383	****	− 0.590	****
Activated memory CD4 T cells	0.052	ns	0.137	***
Follicular helper T cells	0.471	****	0.454	****
Regulatory T cells (Tregs)	0.362	****	0.315	****
Gamma delta T cells	0.278	****	0.562	****
Resting NK cells	− 0.255	****	− 0.503	****
Activated NK cells	0.135	***	0.245	****
Monocytes	− 0.171	****	− 0.350	****
Macrophages M0	0.049	ns	0.069	ns
Macrophages M1	0.183	****	0.222	****
Macrophages M2	− 0.289	****	− 0.375	****
Resting Dendritic cells	− 0.211	****	− 0.264	****
Activated Dendritic cells	− 0.290	****	− 0.375	****
Resting Mast cells	− 0.363	****	− 0.480	****
Activated Mast cells	− 0.015	ns	− 0.060	ns
Eosinophils	− 0.348	****	− 0.380	****
Neutrophils	− 0.271	****	− 0.284	****

^****^p < 0.0001, ***p < 0.001, **p < 0.01, *p < 0.05, p = ns (no significance)

### RUFY4 depletion impaired proliferation rather than migration or invasion of cancer cells

**Fig. 5:**
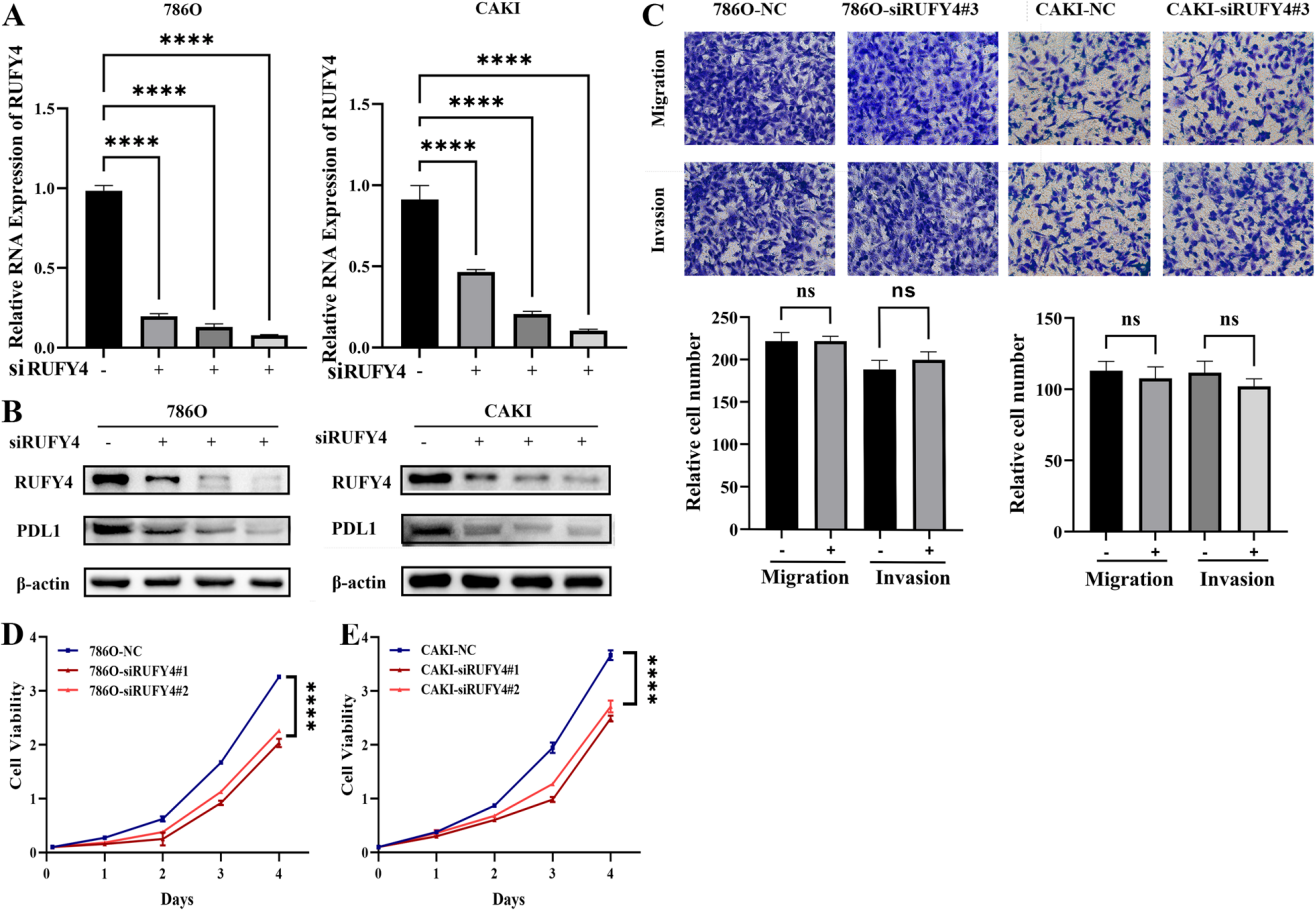
RUFY4 depletion impaired proliferation rather than migration or invasion of cancer cells*. ***A **Relative mRNA levels of *RUFY4* in the control cells and* RUFY4*-knockdown cells. t-test, ****p < 0.0001. **B **Western blot assay for the protein levels of *RUFY4* in indicated cells. **C** Migration and invasion assay for indicated RCC cells (Magnification: 200×). t-test, p = ns (no significance). **D** Cell growth curves of CCK8 assays for indicated cells. t-test, ****p < 0.0001

To ascertain the function of *RUFY4* in TME, small interfering RNAs targeted *RUFY4* were applied to knockdown *RUFY4* in RCC cells, 786O and CAKI. As was shown in Fig. 5A and B, RUFY4 was silenced in RCC cell lines both in RNA and protein levels. And Fig. 5D, E demonstrated *RUFY4* depletion impaired cell proliferation. Our previous results showed that *RUFY4* is related to the distant metastasis and lymphatic metastasis in cancer patients (shown in the previous Fig. 4O, P). Here, we found that down-regulated *RUFY4* had no influence on cell migration and invasion (Fig. 5C). These results hinted that *RUFY4* itself has no significant role in the progress of ccRCC. More in-depth research needs to be carried out to explain these contradictory results.

### RUFY4 predicted immunotherapy in a PDL1-related manner

**Fig. 6:**
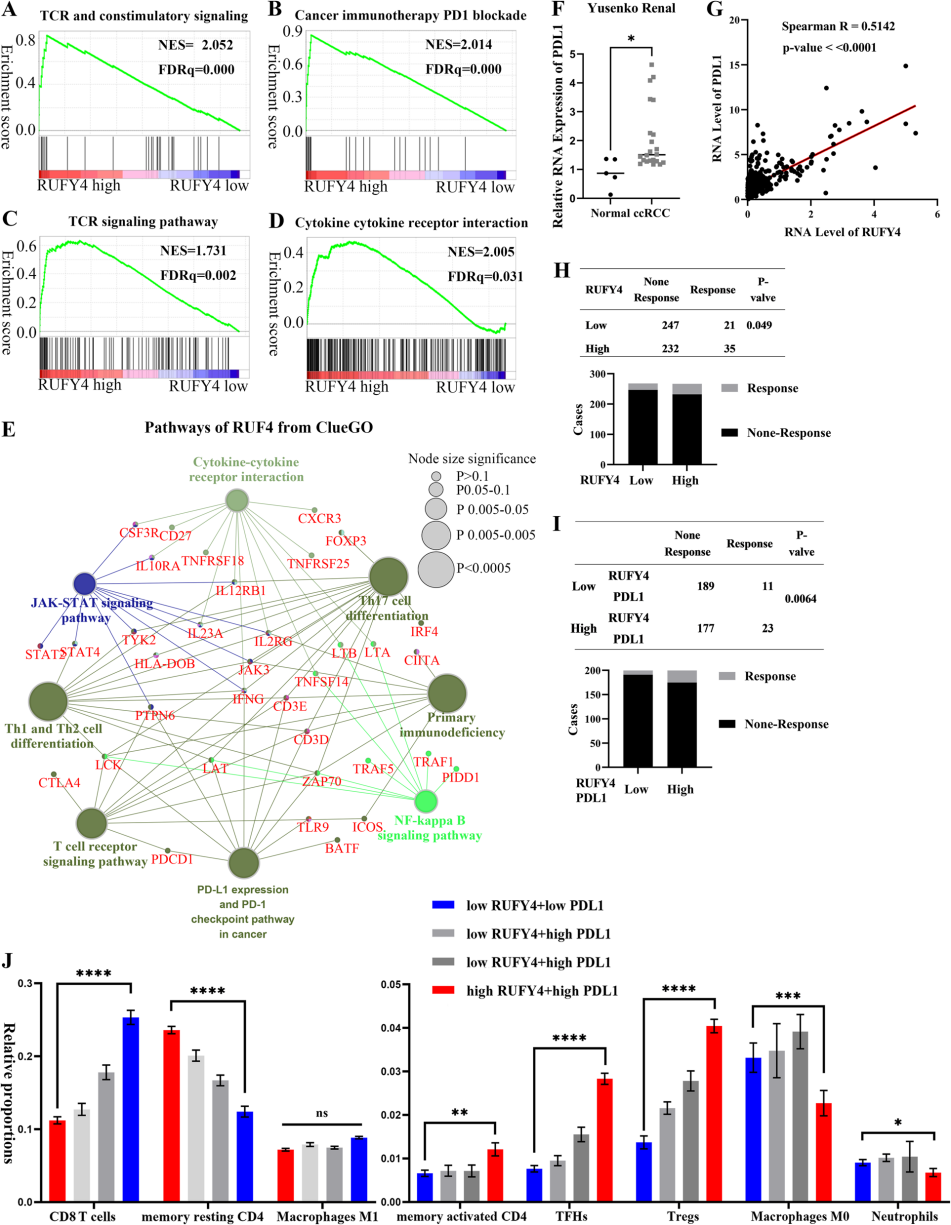
RUFY4 predicted immune infiltration and immunotherapy in a PDL1 related manner*. ***A**–**D **GSEA for the correlation between *RUFY4* and TCR signaling pathway, cancer immunotherapy by PD-1 blockades, TCR and costimulatory signaling, and TCR costimulatory signaling. FDR < 25%, p < 0.05 was considered statistically significant. **E **Pathway network diagram established by clueGO based on the co-expressed genes of *RUFY4*. The color of each circle represents one type of signal pathway and its size represents the degree of statistical significance. Genes involved in each signal pathway are marked with red labels. **F **The mRNA levels of *PDL1* in 22 ccRCC tissues and 5 adjacent normal tissues in ccRCC based on data from the Oncomine database. t-test, *p < 0.05.** G **The relationship between the expression level of *RUFY4* and *PDL1* based on the data from TCGA database. Spearman-test, p < 0.0001.** H **The relationship between the expression level of *RUFY4* and the sensitivity of ICB immunotherapy. Chi-square tests were used to calculate the p-valve. The cut-off for classifying a patient into High/Low expression is the median of expression level. **I **The relationship between the expression level of *RUFY4-PDL1 *and the sensitivity of ICB immunotherapy. Low *PDL1-RUFY4 *means *RUFY4* and *PDL1* were both lower than the median expression level. Chi-square test were used to calculated the p-valve. **J **The difference of 8 immune cell proportions between low and high *PDL1/RUFY4* groups. Low *PDL1* + *low RUFY4* means *RUFY4* and *PDL1* were both lower than the median of expression level. Chi-square tests were used to calculate the p-valve. **p < 0.01, ***p < 0.001, ****p < 0.0001. p = ns (no significance)

We hypothesized that the immune cells in TME might be involved in the tumor-promotive function of *RUFY4* based on the findings that *RUFY4* functioned little on migration and invasion of ccRCC. Table 2 demonstrated that there were correlations between 17 types of immune cells and *RUFY4*, 6 types of which were positively correlated with *RUFY4* while 11 were negatively correlated. Next, a co-expression network of *RUFY4* was constructed (Additional file 5: Fig. S5C). As expected, the network of *RUFY4* was shown to be involved in several crucial immunomodulation processes, including cytokine-cytokine receptor interaction, *PD-L1* (programmed cell death 1 ligand 1) expression and *PD-1* (programmed death-1) checkpoint pathway in cancer, T helper cells differentiation, T cell receptor signaling pathway, and primary immunodeficiency (Fig. 6E). These results emphasized that the *RUFY4* might have an inseparable connection with immunomodulation in TME of ccRCC. After choosing canonical pathways gene sets derived from the KEGG pathway database as the molecular signatures database (Fig. 6C, D), GSEA indicated that *RUFY4* was highly associated with T cell receptor signaling pathway and cytokine-cytokine receptor interaction. Moreover, as presented in Fig. 6A, B, it was found that when compared with gene sets derived from the WikiPathways pathway database, cancer-immunotherapy with *PD1*-blockade and costimulatory signaling of T cell receptor were the two most significant enrichment pathways, whose q-FDR (false discovery rate) values both equaled to zero. These pieces of evidence indicated that the cancer-promoting effect of *RUFY4* was inseparable from the assistance of immunomodulation in TME. Therefore, *PD-1* (programmed death-1) checkpoint pathway in cancer that repeatedly appeared in the functional enrichment results appealed to this study. Moreover, The RNA-Seq data from Yusenko Renal study showed that *PDL1* was upregulated in ccRCC (Fig. 6F). To explore how *RUFY4* contributed to *PD-1/PDL1* in TME, the correlation analysis between *PDL1* and *RUFY4* was conducted on the basis of the ccRCC RNA-Seq data. There was a strong relationship between them and the spearmen R index equaled 0.5142 (Fig. 6G). Also, small interfering RNA targeted *RUFY4* were applied to degrade the expression level of *RUFY4*. As was shown in previous Fig. 5B, the depletion of *RUFY4* caused the decrease of *PDL1* in RCC cell lines, 786O and CAKI. Furthermore, the expression level of *RUFY4* could predict the responsiveness of tumor patients to ICB immunotherapy (Fig. 6H). Once *PDL1* and *RUFY4* were united together, the prediction model was more convincing and valuable (Fig. 6I). And as for immune infiltration prediction, the joint prediction ability of *PDL1* and *RUFY4* was stronger than the single one (Fig. 6J). Taken together, these observations suggested that *RUFY4* might be an indicator for immune infiltration and immunotherapy in a *PDL1*-related manner.

## Discussion

Clear cell RCC is one of the most lethal types of urogenital tumors with an increasing mortality rate over several years [29]. Since Galon [30] first proposed the concept of immune contexture in 2007, researches associated with the location, density, and functional orientation of different immune cells in the TME have put immunotherapy into the cutting-edge frontier. Among the current therapeutic strategies, ICI has become a key option for various cancer types [31]. Ample clinical trials have shown that PD-1 blockade is a vital method in the management of ccRCC and therapies based on anti-PD-1 are the first-line choices for refractory patients [32, 33]. Hence, we aimed at identifying immune cells and genes closely associated with both immune infiltration and immunotherapy responsiveness in the TME.

In this study, TFHs and Tregs were related to patients’ OS after adjusting for known risk factors. TFHs are specialized T helper (TH) cells and different from other subgroups such as TH1, TH2, and TH17. TFHs predicted improved survival in breast cancer [34] and were associated with a positive prognosis in colorectal cancer [35]. Contrarily, TFHs predicted a negative clinical prognosis in lung squamous cell carcinoma [36]. These results put TFHs in a controversial position and thus further exploration should be performed to determine whether it is a protective factor or a risk factor. Treg is another crucial survival-associated immune cell, which is a widely known restrainer of the immune regulatory network. Tregs are involved in balancing immune responses [37] and act as a double-edged sword. On the one hand, they can restrain unnecessary immune activations, such as autoimmunity. On the other hand, useless immune responses are utilized by Tregs for pathogens or cancer cells, resting in the progression of cancer [38]. In renal cancers, there has been evidence for the association between Tregs and poor prognosis, as well as immunotherapy resistance. In summary, these cells play important roles in the construction of the TME.

Among IIRGs in this study, *RUFY4* is closely related to the dense infiltration of TFHs and Tregs. Although the importance of IIRGs in tumor progression and immunotherapy has been recognized, there are few studies dedicated to genes related to the immune infiltration of TFHs and Tregs. Here, this research innovatively proposes a method of a targeted selection of IIRGs. Additionally, our results denoted that the depletion of *RUFY4* caused the decrease of *PDL1*. Previous studies [39–41] revealed that JAK-STAT axis primarily regulates *PDL1* expression in tumor cells through the activation of transcription pathway. Angel [39] considered that the JAK-STAT3 regulated *PDL1* expression by activation of its promoter. The role and function of the NF-kB signaling pathway cannot be overlooked. Peng [42] found that chemotherapy induces local immune suppression in ovarian cancer through NF-kappa B-mediated *PDL1* upregulation. Fabrizio proposed that NF-kappa B directly induces *PDL1* gene transcription by binding to its promoter, and it can also regulate *PDL1* post-transcriptionally through indirect pathways [43]. Together with our finding that *RUFY4* was also closely linked to JAK-STAT and NF-kappa B pathways, we speculate that *RUFY4* regulated the expression level of *PDL1* by participating in JAK-STAT and/or NF-kappa B signaling pathways.

Another finding in this study is that *RUFY4* depletion has no effect on migration or invasion of cancer cell in vitro while *RUFY4* is related to the distant metastasis and lymphatic metastasis in cancer patients. To explain this conflict phenomenon, we make a hypothesis that the immune system might be involved in the tumor-promotive function of *RUFY4* based on the following evidence: (a) Metastasis is under the control of complex and redundant pathways involving the tumor cell and the microenvironment [44, 45]. More and more evidence confirms that a large array of genes is known to facilitate or regulate cancer metastasis, not just a single molecular effect [44, 46–50]; (b) TME represents the necessary prerequisite for cancer progression and metastasis [51–53]; (c) Tregs [54] and TFHs [55] control cancer invasion and migration because they might establish an immunosuppressive microenvironment within primary lesions; and (d) *RUFY4* is considered an immunological biomarker of THFs and Tregs, and its depletion contributes to the reduction of *PDL1*. Thus, we guess that the depletion of *PDL1* could be recognized by THFs or Tregs, which might be responsible for *RUFY4*-mediated cancer metastasis. There is no doubt that more in-depth mechanism research needs to be carried out in the future.

## Conclusion

The present study analyzed and verified a unique gene *RUFY4* closely related to immune infiltration of TFHs and Tregs. These cells and the gene were separately recognized as independent prognostic biomarkers of ccRCC patients. Moreover, *RUFY4* could predict the responsiveness of ICI therapy for ccRCC patients in a *PDL1*-related manner. Our findings also provide strategies to explore effective signatures of immunotherapy and clinical outcome in cancer research.

## Supplementary Information

**Additional file 1: Figure S1.** Flowchart of data processing

**Additional file 2: Figure S2. A–I** The univariate survival analysis for the relative proportions of the nine immune cells. The red line indicates a high proportion group of immune cells, and the blue line indicates a low proportion group of immune cells. The cut-off for classifying a patient into High/Low is the median of relative proportion. Long-rank tests were used for statistics. P-valves were on Figures.

**Additional file 3: Figure S3. A–G** The relationship between the relative proportions of immune cells and pathological stage and clinical stage. The ordinate represents the proportions of immune cells and the horizontal line inside the box represents the median value of immune cell proportions. Kruskal-test and Wilcoxon-test were used in statistical analysis. P-valves were on Figures.

**Additional file 4: Figure S4. A–D** The enrichment analysis results of IIRGs at biological processes, cellular components, molecular functions, and KEGG levels. The top 10 results of each term are shown and IIRGs is the abbreviation of immune-infiltration-related genes.

**Additional file 5: Figure S5. A** The multivariate survival analysis for the expression level of LAG3. The red line indicates a high expressing group of LAG3, and the blue line indicates a low expressing group. HR means hazard ratio and CI means confidence interval. P-valve was on the Figure. **B** The histological expression of *RUFY4* from Human Protein Atlas. **C** Verifying the correlations between genes in the co-expression network of *RUFY4*. The size of each dot represents the statistical significance and its color means the correlation index.

## Data Availability

The datasets analyzed during the current study are available in the TCGA repository (https://portal.gdc.cancer.gov), Gene Expression Omnibus (GEO) at GSE126864 and Oncomine repository. Other data generated in this study are available upon request from the corresponding author.

## Abbreviations

IIRG: Immune-infiltrated-related gene
TFHs: Follicular helper T cells
Tregs: Regulatory T cells
RUFY: RUN and FYVE domain–containing protein
PI3P: Phosphatidylinositol 3-phosphate
PODXL: Podocalyxin-like
TCGA: The Cancer Genome Atlas Project
KM: Kaplan–Meier
GO: Gene ontology
KEGG: Kyoto Encyclopedia of Genes and Genomes
PD-L1: Programmed cell death 1 ligand 1
PD-1: Programmed death-1
ccRCC: Clear cell Renal cell carcinoma
ICI: Immune checkpoint inhibitors
TME: Tumor microenvironment
DMEM: Dulbecco’s modified eagle medium
