# Supplementary figures and images for: As a prognostic biomarker of clear cell renal cell carcinoma RUFY4 predicts immunotherapy responsiveness in a PDL1-related manner
Source: Cancer Cell Int. 2022 Feb 8;22:66. doi: 10.1186/s12935-022-02480-7 (PMC8822671; doi:10.1186/s12935-022-02480-7)

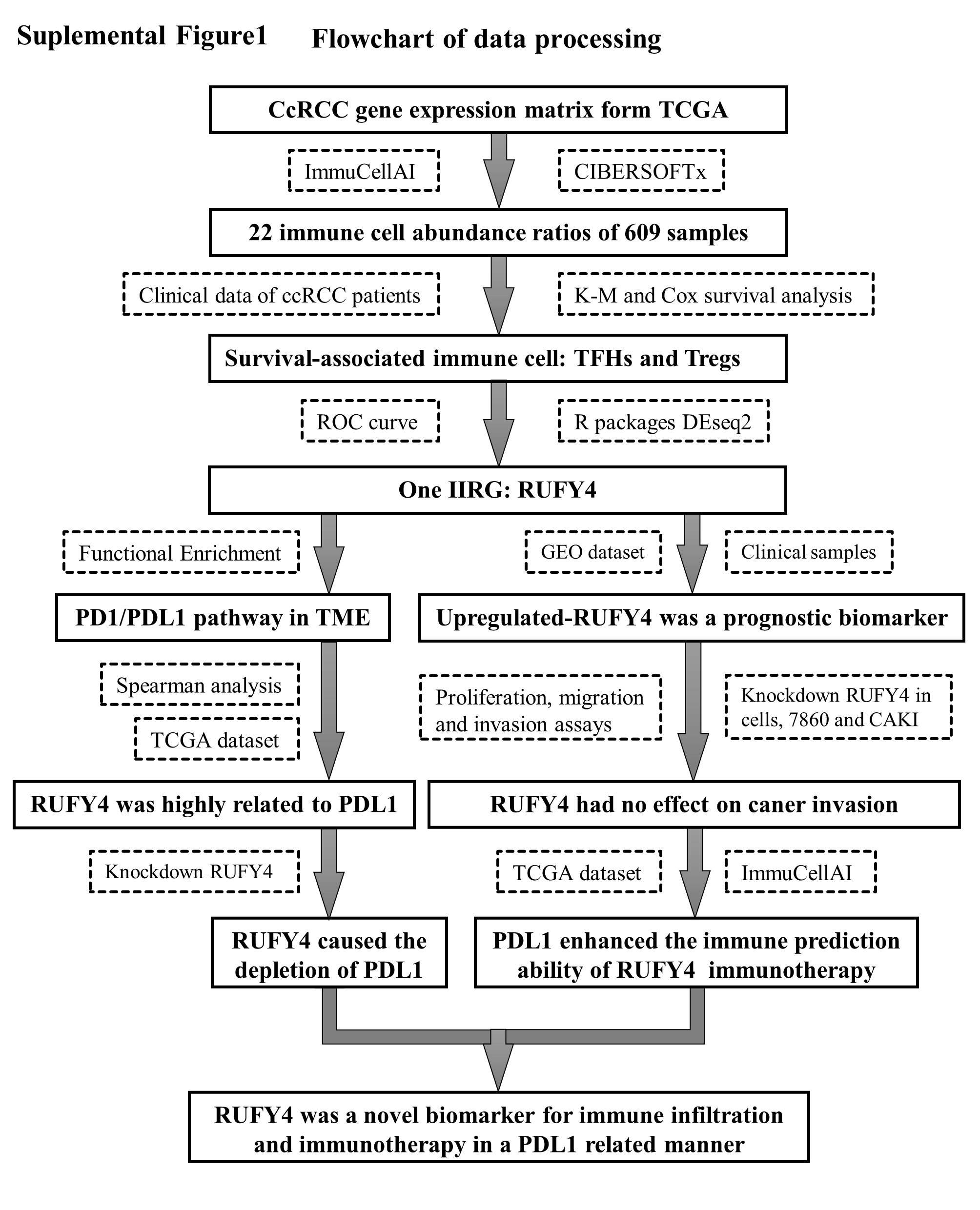

Supplement: Supplementary file 1 — Additional file 1: Figure S1. Flowchart of data processing [file 12935_2022_2480_MOESM1_ESM.tif]

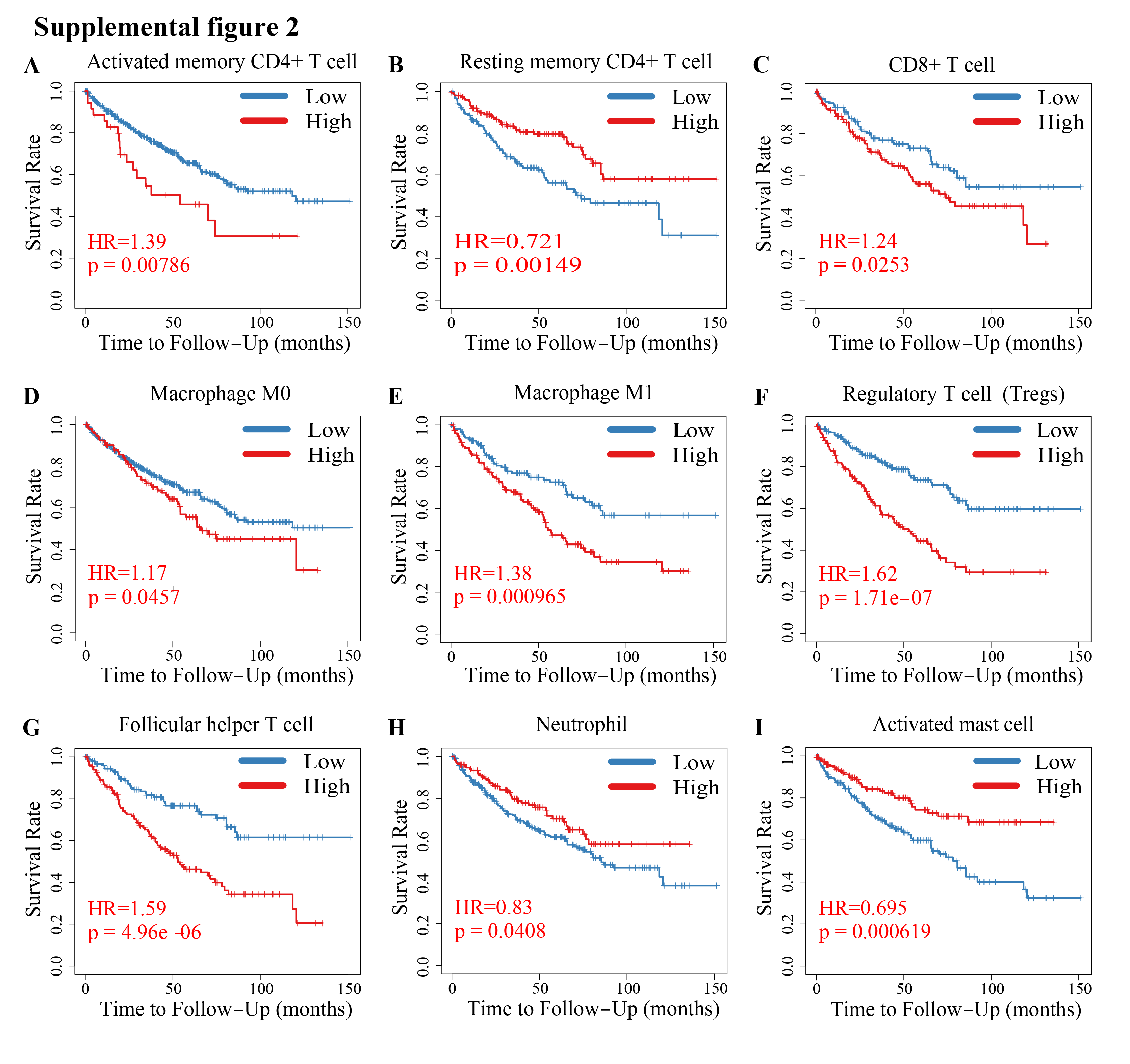

Supplement: Supplementary file 2 — Additional file 2: Figure S2. A–I The univariate survival analysis for the relative proportions of the nine immune cells. The red line indicates a high proportion group of immune cells, and the blue line indicates a low proportion group of immune cells. The cut-off for classifying a patient into High/Low is the median of relative proportion. Long-rank tests were used for statistics. P-valves were on Figures. [file 12935_2022_2480_MOESM2_ESM.tif]

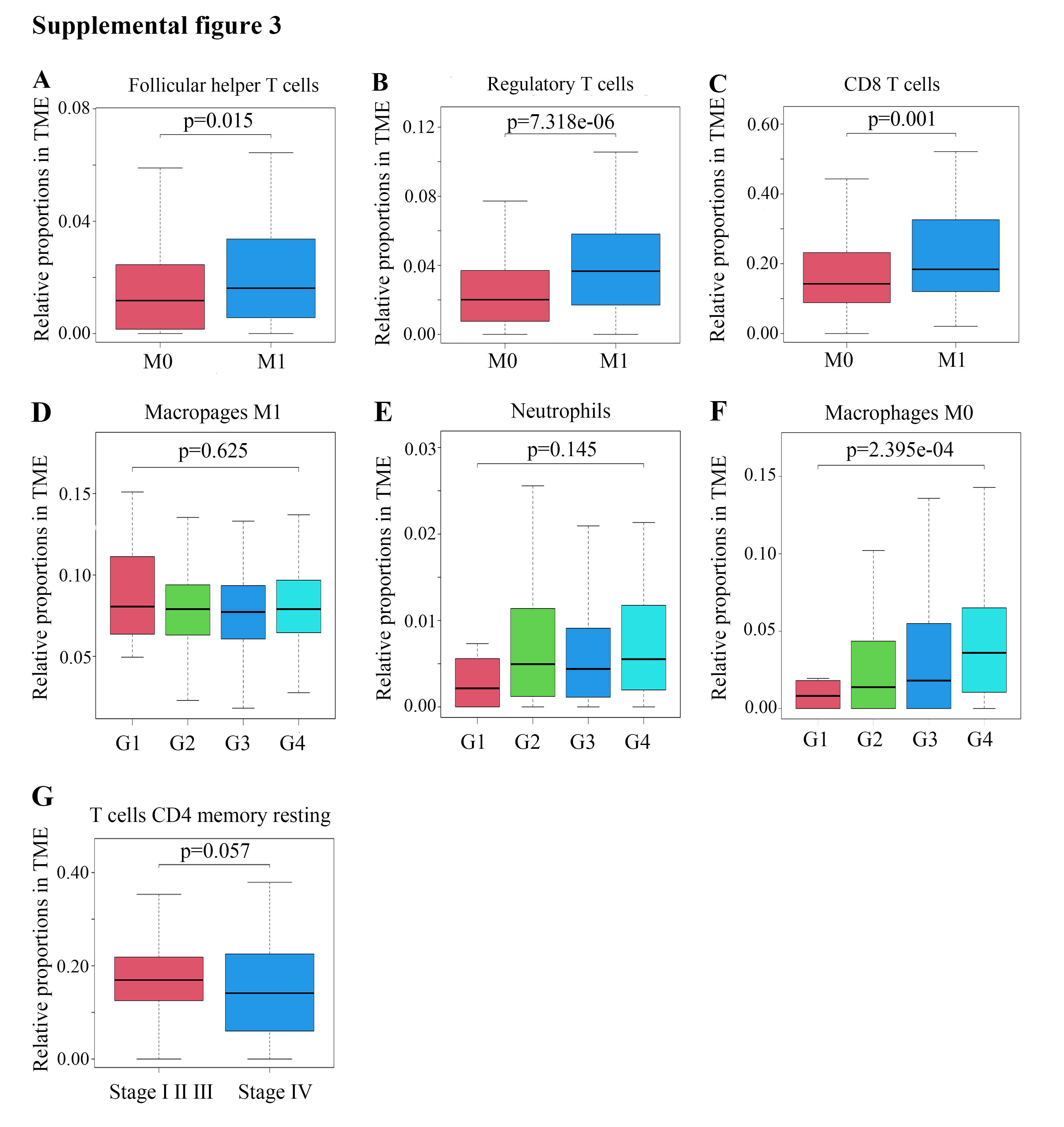

Supplement: Supplementary file 3 — Additional file 3: Figure S3. A–G The relationship between the relative proportions of immune cells and pathological stage and clinical stage. The ordinate represents the proportions of immune cells and the horizontal line inside the box represents the median value of immune cell proportions. Kruskal-test and Wilcoxon-test were used in statistical analysis. P-valves were on Figures. [file 12935_2022_2480_MOESM3_ESM.tif]

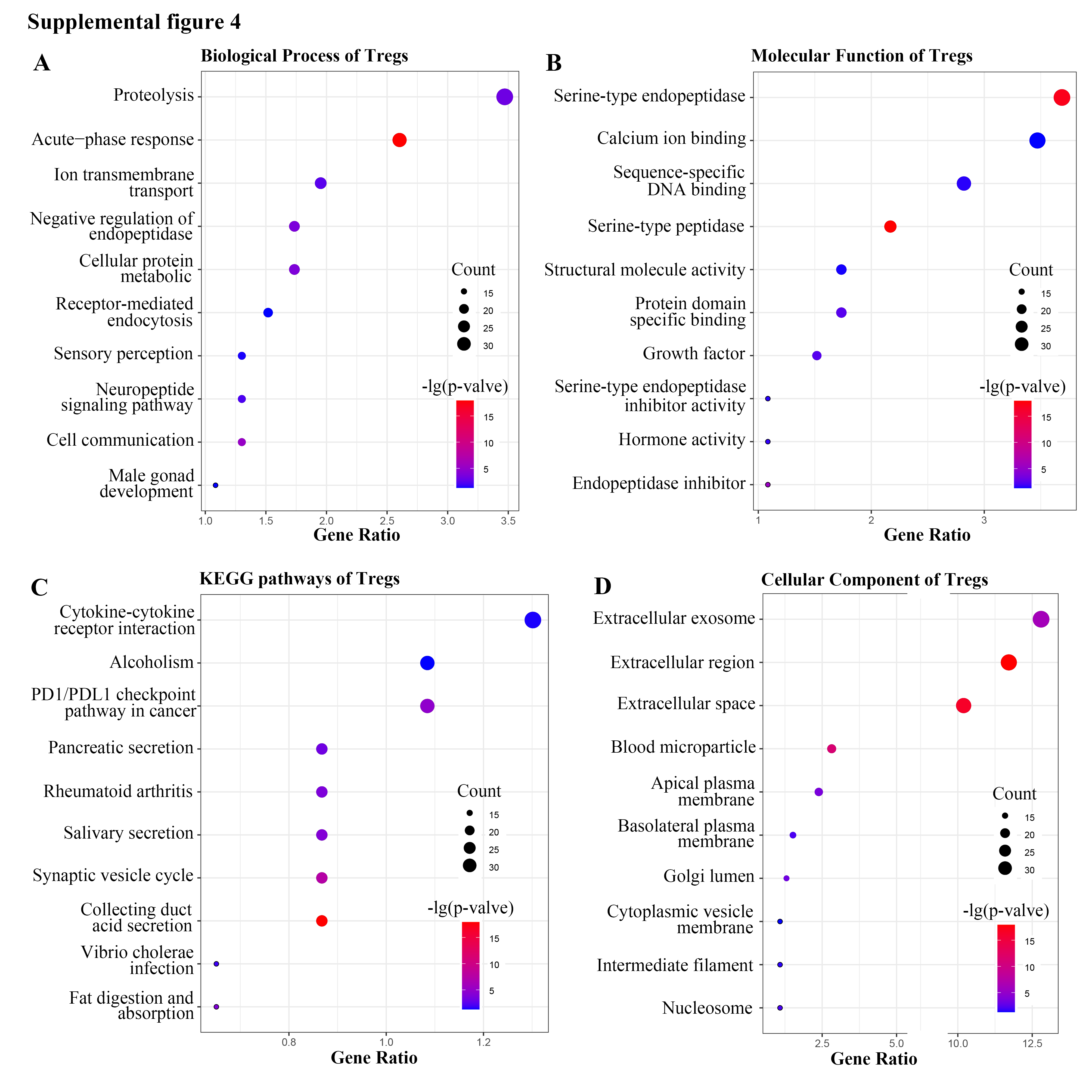

Supplement: Supplementary file 4 — Additional file 4: Figure S4. A–D The enrichment analysis results of IIRGs at biological processes, cellular components, molecular functions, and KEGG levels. The top 10 results of each term are shown and IIRGs is the abbreviation of immune-infiltration-related genes. [file 12935_2022_2480_MOESM4_ESM.tif]

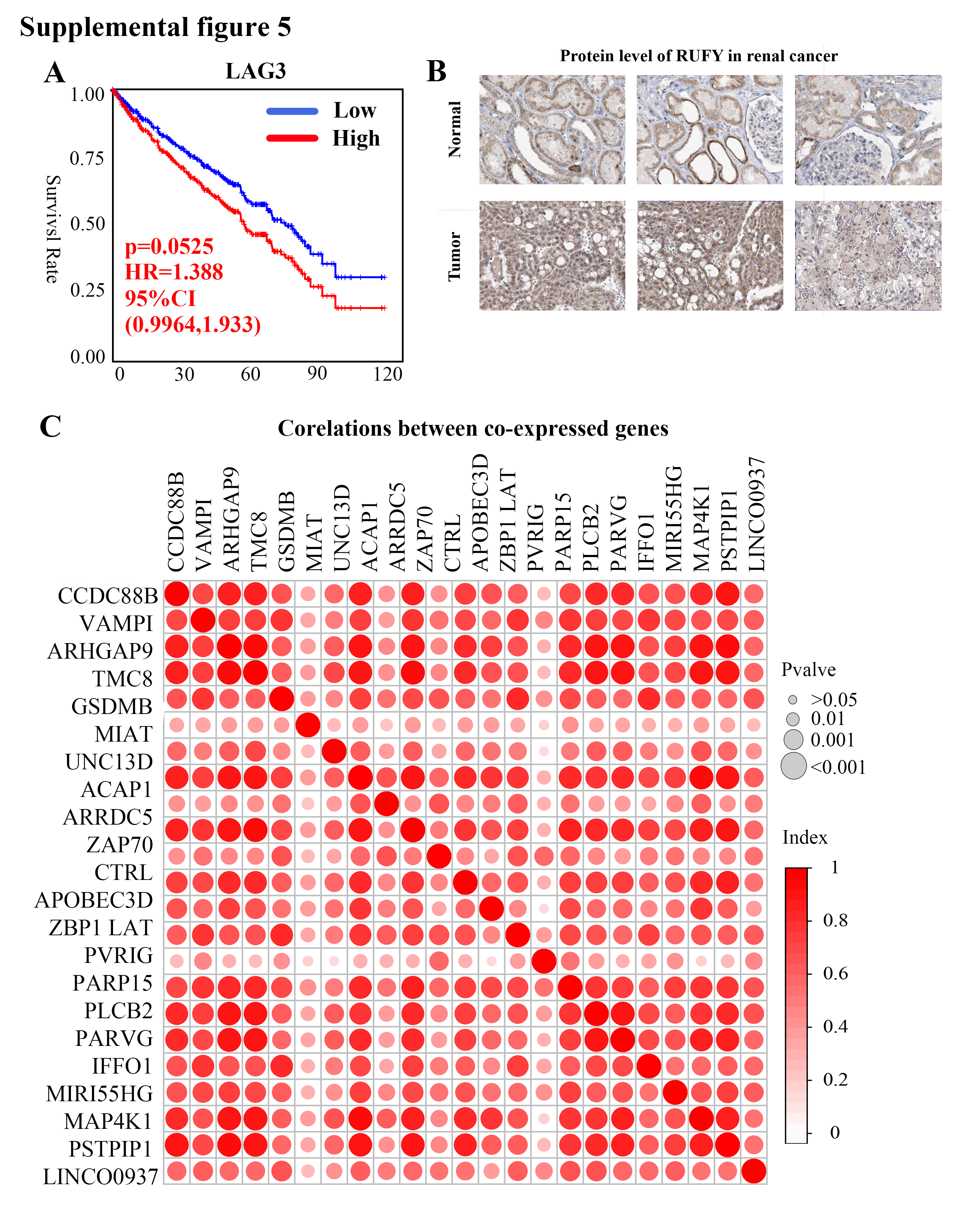

Supplement: Supplementary file 5 — Additional file 5: Figure S5. A The multivariate survival analysis for the expression level of LAG3. The red line indicates a high expressing group of LAG3, and the blue line indicates a low expressing group. HR means hazard ratio and CI means confidence interval. P-valve was on the Figure. B The histological expression of RUFY4 from Human Protein Atlas. C Verifying the correlations between genes in the co-expression network of RUFY4. The size of each dot represents the statistical significance and its color means the correlation index. [file 12935_2022_2480_MOESM5_ESM.tif]
